# Moderate organic fertilizer substitution for partial chemical fertilizer improved soil microbial carbon source utilization and bacterial community composition in rain-fed wheat fields: current year

**DOI:** 10.3389/fmicb.2023.1190052

**Published:** 2023-06-15

**Authors:** Xiaoli Liu, Wenping Yang, Wenguang Li, Aamir Ali, Jie Chen, Min Sun, Zhiqiang Gao, Zhenping Yang

**Affiliations:** ^1^College of Agriculture, Shanxi Agricultural University, Taigu, Shanxi, China; ^2^College of Life Sciences, North China University of Science and Technology, Tangshan, China

**Keywords:** organic manure partial substitution chemical fertilizer, soil microbial carbon sources metabolic characteristics, soil bacteria community, bacteria functional prediction, soil nutrients, wheat yield

## Abstract

Organic fertilizers can partially replace chemical fertilizers to improve agricultural production and reduce negative environmental impacts. To study the effect of organic fertilizer on soil microbial carbon source utilization and bacterial community composition in the field of rain-fed wheat, we conducted a field experiment from 2016 to 2017 in a completely randomized block design with four treatments: the control with 100% NPK compound fertilizer (N: P_2_O_5_: K_2_O = 20:10:10) of 750 kg/ha (CK), a combination of 60% NPK compound fertilizer with organic fertilizer of 150 kg/ha (FO1), 300 kg/ha (FO2), and 450 kg/ha (FO3), respectively. We investigated the yield, soil property, the utilization of 31 carbon sources by soil microbes, soil bacterial community composition, and function prediction at the maturation stage. The results showed that (1) compared with CK, organic fertilizer substitution treatments improved ear number per hectare (13%−26%), grain numbers per spike (8%−14%), 1000-grain weight (7%−9%), and yield (3%−7%). Organic fertilizer substitution treatments increased the total nitrogen, available nitrogen, available phosphorus, and soil organic matter contents by 26%, 102%, 12%, and 26%, respectively, compared with CK treatments. Organic fertilizer substitution treatments significantly advanced the partial productivity of fertilizers. (2) Carbohydrates and amino acids were found to be the most sensitive carbon sources for soil microorganisms in different treatments. Particularly for FO3 treatment, the utilization of β-Methyl D-Glucoside, L-Asparagine acid, and glycogen by soil microorganisms was higher than other treatments and positively correlated with soil nutrients and wheat yield. (3) Compared with CK, organic fertilizer substitution treatments increased the relative abundance of Proteobacteria, Acidobacteria, and Gemmatimonadetes and decreased the relative abundance of Actinobacteria and Firmicutes. Interestingly, FO3 treatment improved the relative abundance of *Nitrosovibrio, Kaistobacter, Balneimonas, Skermanella, Pseudomonas*, and *Burkholderia* belonging to Proteobacteria and significantly boosted the relative abundance of function gene K02433 [the aspartyl-tRNA (Asn)/glutamyl-tRNA (Gln)]. Based on the abovementioned findings, we suggest FO3 as the most appropriate organic substitution method in rain-fed wheat fields.

## 1. Introduction

Fertilization is crucial for modern agricultural production. Fertilizer types and application rates greatly affect crop production, soil properties, and soil microorganisms (Geisseler et al., 2017). The application of chemical fertilizers can increase the yield of crops by more than 50% (Tang et al., 2020). However, in the past 20 years, with the substantial amount of chemical fertilizer application, the phenomenon that its returns were diminishing appeared in agricultural production (Peng et al., 2021), especially in the arid areas on the Loess Plateau with insufficient precipitation and poor soil texture and quality. Excessive chemical fertilizer aggravates soil acidification, hardening, and nutrient loss, thereby reducing crop yield (Hui et al., 2018; Li, 2021).

Fertilization has significant effects on the activity of soil microbes (Francioli et al., 2016). Fertilizer application also influences the evolutions in the soil pH, available potassium, SOC, total N, and microbial biomass C and N, which were observed upon fertilizer application (Ji et al., 2018). The application of organic fertilizer with rich nutrients and a slow-release rate can not only alleviate the deterioration of soil physiochemical and biological properties caused by chemical fertilizer inputs but also supply various nutrients for crop growth, promote microbial reproduction, and enhance the ability of soil to retain fertilizer and water (Karuku et al., 2018). Previous studies have revealed that the combined application of organic fertilizer and chemical fertilizers significantly increased the crop yield and reproduction of soil microorganisms (Dominchin et al., 2021; Han et al., 2021a), as well as improved soil fertility and soil bacterial community composition (Wang et al., 2021). Soil microbiota plays key roles in regulating the soil micro-ecological environment, promoting the uptake of nutrient elements in roots, and maintaining soil fertility and sustainability (Li et al., 2022). The higher proportion of organic fertilizer increased soil bacterial diversity (Liu et al., 2021).

Recent studies on high-throughput sequencing have provided new insights into soil microbial diversity and community composition under long-term organic and inorganic fertilization. However, (1) How does soil microbial community composition change as the proportion of organic fertilizer instead of chemical fertilizer increases? (2) What is the soil microbial carbon source utilization under the organic substitution treatments? (3) How is the relationship between the soil microbial community composition and carbon source utilization? and (4) What is the best organic substitution treatment? To answer these questions, we measured the relevant parameters of soil nutrients, investigated the utilization of soil microbial carbon sources using the Biolog-eco microplate method, and analyzed bacterial community structure using 16s rRNA gene Illumina MiSeq high-throughput sequencing under different substitution proportions of organic fertilizer for partial chemical fertilizer in a rain-fed wheat field. The differential analysis and correlation analysis were used to reveal the potential effects of the community composition of specific bacterial taxa on carbon source utilization capacity, functional genes, and their association with soil nutrients. The study results provided useful information for improving the quality and efficiency of agricultural production as well as reducing the negative impact of fertilizers on the environment.

## 2. Materials and methods

### 2.1. Study site description

The experiment was conducted from 2016 to 2017 in Yuanqu County (35°14.4′N, 111°43.3′E), Shanxi Province, which is located on the Loess Plateau in northwest China. This region has a sub-humid, warm, and continental monsoon climate with a mean frost-free period of 236 days and an average annual precipitation, temperature, and sunshine time of 631 mm, 13.5°C, and 2 026.2 h, respectively. The soil texture is medium loam and classified as cinnamon red vertical structural loess (Cui et al., 2018; Hui et al., 2018). Before sowing on 30 September 2016, the soil properties in the study field were recorded as follows: pH of 8.0, 10.5 g kg^−1^ soil organic matter (SOM), 0.71 g kg^−1^ total nitrogen (TN), 86.3 mg kg^−1^ alkali-hydrolyzable nitrogen N (AN), 14.5 mg kg^−1^ available phosphorus (AP), and 112.3 mg kg^−1^ available potassium (AK) in the 0–20 cm deep soil layer.

### 2.2. Experimental design and treatments

The field experiment was performed in a completely randomized block design with three biological replicates for each treatment, including the control with 100% chemical fertilizer of 750 kg/ha (CK), a combination of 60% chemical fertilizer with organic fertilizer of 150 kg/ha (FO1), 300 kg/ha (FO2), and 450 kg/ha (FO3), respectively (Table 1). There were 12 plots, and the plot size was 660 m^2^ (15 m × 44 m). Before sowing winter wheat (cultivar Yannong-21; 112.5 kg/ha) on 30 September 2016, we applied organic fertilizer with a depth of 40–60 cm in the soil. Chemical fertilizer was applied to all plots at the time of sowing using the wide ridge and narrow furrow sowing methods with wide ridges (with a 25-cm wide base and 12 cm height) and narrow furrows (depth 8 cm, sown into the top edges of the furrow, rows spaced 12 cm). Other field management measures, such as irrigation and weeding, were carried out routinely. The organic fertilizer (organic matter ≥45%, N: 2.5%, P_2_O_5_: 1.4%, and K_2_O: 1.6%) and chemical fertilizer (NPK compound fertilizer, N: P_2_O_5_: K_2_O = 20:10:10) were purchased from Guilong Co., Ltd., China.

**Table 1:** Experiment treatments and fertilization methods.

**Treatments**	**Application of chemical fertilizer (chemical NPK) (kg/ha)**	**Proportion (%)**	**Reduction of chemical fertilizer (chemical NPK) (kg/ha)**	**Proportion (%)**	**Replacement of organic fertilizer (organic matter + organic NPK) (kg/ha)**	**Proportion (%)**
CK	750	100	0	100	0	0
FO1	450	60	300	40	150	20
FO2	450	60	300	40	300	40
FO3	450	60	300	40	450	60

### 2.3. Sampling and chemical analysis

The wheat was harvested and yield tested on 8 June 2017. After the harvest, all the wheat in each plot was threshed and weighed. To determine the grain moisture content, we used a fast moisture meter (PM-8188 New, Kett, Japan; three times in each plot). According to the standard moisture content (13%), the actual yield of each plot was obtained.

Soil samples were collected before harvesting. Three sampling sites with 1 m^2^ were chosen randomly in each plot, and five subsamples from each site were collected at a depth of 0–20 cm using a sterilized 4 cm-diameter soil-drilling sampler, respectively. After sieving through a 2-mm mesh to filter out roots, large rocks, and other impurities, five subsamples were mixed into one sample and divided into three parts for subsequent analyses. One part (fresh soil) was placed in a 50-ml centrifuge tube and then kept at −80°C for microbial sequencing analysis. One part (fresh soil) was stored at 4°C for soil microbial carbon utilization. The last part was air-dried and used to determine the soil's chemical properties.

The SOM, TN, and AN contents were determined using the potassium dichromate volumetric method, the semi-micro Kjeldahl method, and the alkaline hydrolysis diffusion method (He et al., 2022), respectively. The AP content was determined in a 0.5 mol L^−1^ NaHCO_3_ extraction using the molybdenum-antimony anti-spectrophotometric method (Sun et al., 2019). The AK content was determined in a 1.0 mol L^−1^ NH_4_OAc extraction using leaching-flame photometry (Bell et al., 2005).

### 2.4. Soil DNA extraction, quantitative real-time PCR (q-PCR), and Illumina sequencing

Microbial DNA was extracted from the frozen soil samples (0.5 g wet weight) using a Fast DNA SPIN Extraction Kit (MP Biomedicals, Santa Ana, CA, USA) following the manufacturer's instructions. DNA quality was determined using a NanoDrop2000 Spectrophotometer (Thermo Fisher Scientific, Wilmington, DE, USA). The primer pairs 338F (5′-ACTCCTACGGGAGGCAGCA-3′) and 806R (5′-GGACTACHVGGGTWTCTAAT-3′) targeting the bacterial 16S rRNA gene V3-V4 hypervariable region were used for polymerase chain reaction (PCR) amplification (Langille et al., 2013). The sample-specific 7-bp barcodes were incorporated into the primers for multiplex sequencing. The PCR mixtures included FastPfu buffer (5 ×), primer (5 μM), dNTP mixture (2.5 mM), template DNA, and H_2_O for a total volume of 50 μl. PCR assays were performed as follows: 95°C for 2 min, followed by 30 cycles at 95°C for 30 s, 55°C for 30 s, and 72°C for 1 min. PCR products were quantified and pooled in equimolar concentrations, and the paired-end reads (250 nucleotides) were generated using an Illumina HiSeq 2500 platform at Personal Biotechnology Co., Ltd., Shanghai, China.

The raw sequencing data were subjected to quality filtering and double-terminal sequence ligation using QIIME2. The data filtering were described previously (Peng et al., 2021): removal of sequences containing Ns (fuzzy bases or base mismatch number >1 in the 5′-terminal primer matching); removal of sequences containing the same consecutive base number >8; removal of sequences shorter than 150 bp. The UCLHIME in MOTHUR software (version 1.31.2, http://www.mothur.org/) was used to obtain high-quality sequences for subsequent analysis. The UCLUST method in QIIME was used to cluster the high-quality sequences at 97% sequence identity, and the longest sequence in each class was selected as the representative sequence. The Blast method in QIIME was used to align the Green Genes database (release 13.8, http://greengenes.secondgenome.com/) to obtain the taxonomic information of each OTU (operational taxonomic unit). The OTUs with abundances < 0.001% of the total sequences were removed to obtain a simplified OTU list for subsequent analysis. PICRUSt2 (Phylogenetic Investigation of Communities by Reconstruction of Unobserved States) was used for bacterial function prediction (Russo et al., 2023); the closed OTU table was obtained from QIIME and compared to the KEGG (Kyoto Encyclopedia of Genes and Genomes) database to determine different bacterial community functions.

### 2.5. Carbon source utilization by soil microorganisms

The carbon source utilization ability of soil microorganisms was estimated using the average well-color development (*AWCD*) and determined using the Biolog-ECO microplate method (Chen et al., 2017). The procedure involved weighing a fresh soil sample equivalent to 5 g of dry soil into a sterile conical flask. Then, 45 ml of 0.85% sterile NaC1 solution was added, followed by sealing and shaking at 2,000 r/min for 15 min. The supernatant was collected after letting the solution stand for 15 min. Moreover, the supernatant was diluted 100-fold with sterile NaCl solution on a clean bench and inoculated into Biolog-eco microplates with an 8-channel sample applicator at 150^−1^ μl per well. Inoculated plates were incubated in a 25°C biochemical incubator for 10 days, and the good absorbance (*OD*) at 590 nm was read every 24 h during the incubation period. The absorbance was measured according to the following equation:


$$\begin{array}{l} AWCD\;=\;\left[\sum \left(Ci\;-\;R\right)\right]/n, \end{array}$$

where *C*i is the *OD* value of each carbon source well, *R* is the *OD* value of the control well, and *n* is the number of carbon source types in the culture medium (31 in this study).

### 2.6. Statistical analyses

Origin (2019) was used to plot the line chart of microbial characteristics. SPSS (SPSS 19.0, SPSS Inc., Chicago, USA) was used for variance analysis, principal component analysis (PCA), Spearman correlation analysis (*P*-value), and cluster analysis. The relationship between soil microbial characteristics and soil properties was determined using Spearman correlation analysis. One-way analysis of variance (ANOVA) was used to analyze the different organic substitution patterns for significant differences (*P* < 0.05), such as soil physical and chemical properties and microbial characteristics. The difference between the means was represented with different letters by using the least significant difference (LSD; *P* < 0.05).

## 3. Results

### 3.1. Rain-fed wheat yield characteristics, soil chemical properties, and partial productivity of fertilizers

Crop yield and soil fertility depend on the contents of the main nutrients in the soil, which are largely determined by fertilization. The treatments of organic fertilizer substitution for partially chemical fertilizer significantly affected soil nutrient contents, wheat yield, and yield components in rain-fed wheat fields (Table 2). Compared with the control (CK), the organic fertilizer substitution treatments increased the TN, AN, AP, and SOM by 1.33%−41.33%, 17.73%−147.19%, 7.76%−12.30%, and 6.10%−46.15%, respectively. Organic fertilizer substitution treatments also increased ear number, grain numbers per spike, and 1,000-grain weight by 13.21%−26.00%, 8.33%−11.11%, and 8.09%-9.36%, respectively. Therefore, grain yield was increased by 3.45–6.92%.

**Table 2:** Soil fertility and the economic characteristics of wheat during wheat maturity in rain-fed wheat field under different treatments.

**Treatments**	**Ear number (10^4^/ha)**	**Number of grains per spike**	**1,000 grain weight (g)**	**Yield (kg/ha)**
CK	477 ± 3Cc	36 ± 2Ab	33.86 ± 1.16Bb	5337.34 ± 170.31Ab
FO1	540 ± 2Bb	39 ± 2Aab	36.63 ± 0.77Aa	5521.49 ± 258.34Aab
FO2	546 ± 9Bb	41 ± 4Aa	36.36 ± 0.94ABa	5503.61 ± 322.35Aab
FO3	601 ± 7Aa	40 ± 1Aa	37.03 ± 0.08Aa	5706.45 ± 262.97Aa
	**TN (g/kg)**	**AN (mg/kg)**	**AP (mg/kg)**	**SOM (g/kg)**
CK	0.75 ± 0.01Bc	15.34 ± 0.90Cc	14.55 ± 0.75Ab	7.54 ± 0.23Cd
FO1	0.76 ± 0.02Bc	18.06 ± 0.01Bb	15.68 ± 0.38Aab	8.00 ± 0.01Cc
FO2	1.01 ± 0.01Ab	37.02 ± 0.90Aa	16.91 ± 0.28Aa	9.51 ± 0.35Bb
FO3	1.06 ± 0.03Aa	37.92 ± 0.01Aa	16.34 ± 1.23Aa	11.02 ± 0.01Aa

Different lowercase letters in the same column indicate differ significantly at the 0.05 level (P < 0.05), while different capital letters indicate differ extremely significantly at the 0.01 level (P < 0.01). CK, control; FO1, combination of 60% chemical fertilizer with organic manure of 150 kg/ha; FO2, combination of 60% chemical fertilizer with organic manure of 300 kg/ha; FO3, combination of 60% chemical fertilizer with organic manure of 450 kg/ha. TN, total nitrogen; AN, available nitrogen; AP, available phosphorus; SOM, soil organic matter.

Organic fertilizer treatments for the soil fertility index significantly increased soil AN, TN, SOM, and AP content, respectively, compared to CK (Table 2). This fact indicates that the partial replacement of chemical fertilizer with organic fertilizer is beneficial for the transformation and accumulation of soil N/C/P nutrients.

No significant differences were found between FO2 and FO3 in yield or the values of AN and AP. The results showed that an equal amount of substitution (40%) and an increased substitution (60%) of organic fertilizer may improve the soil chemical properties at a stable wheat yield level in rain-fed.

Compared with CK, the organic substitution treatment significantly increased the partial productivity of N (143%), P (131%), and K (128%) fertilizers, respectively (Table 3).

**Table 3:** Inorganic fertilizer application amount and fertilizer partial productivity of different treatments.

**Treatments**	**Application amount of inorganic fertilizer (kg/ha)**			**Partial productivity/(kg**·**kg**^**−**^**1)**		
	**N**	**P2O5**	**K2O**	**N**	**P**	**K**
CK	150	75	75	35.58 ± 1.14Cc	71.16 ± 2.27Cc	71.16 ± 2.27Cc
FO1	60	30	30	86.61 ± 4.05Aa	172.01 ± 8.05Aa	170.42 ± 7.97Aa
FO2	60	30	30	81.53 ± 4.78Aab	160.92 ± 9.43Aa	158.15 ± 9.26Ab
FO3	60	30	30	80.09 ± 3.69Bb	157.20 ± 7.24Bb	153.40 ± 7.07Bb

Different lowercase letters in the same column indicate differ significantly at the 0.05 level (P < 0.05), while different capital letters indicate differ extremely significantly at the 0.01 level (P < 0.01). CK, control; FO1, combination of 60% chemical fertilizer with organic manure of 150 kg/ha; FO2, combination of 60% chemical fertilizer with organic manure of 300 kg/ha; FO3, combination of 60% chemical fertilizer with organic manure of 450 kg/ha. TN, total nitrogen; AN, available nitrogen; AP, available phosphorus; SOM, soil organic matter.

### 3.2. Soil carbon source utilization by soil microorganisms

**Figure 1:**
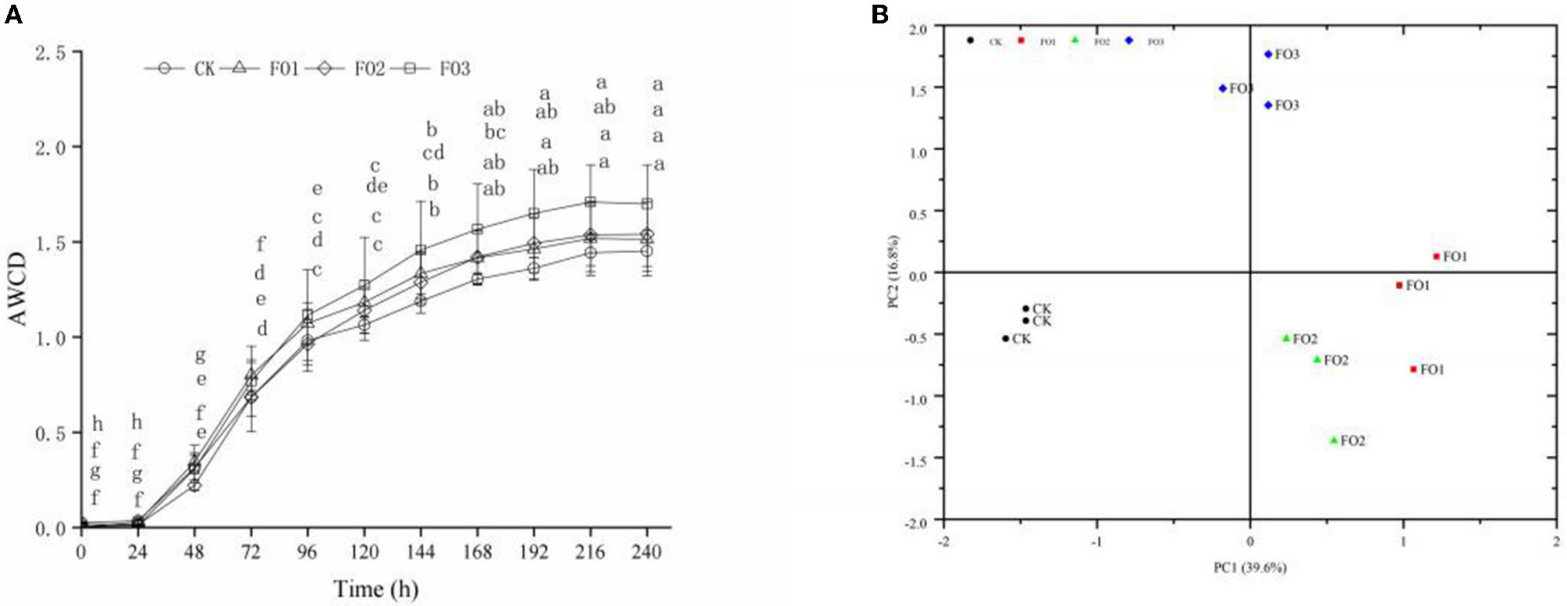
Average well color development *(AWCD)* **(A)** and principal component analysis **(B)** of carbon sources utilization profiles of soil microbes in Biolog Ecoplate of soil samples under different treatments. **(A)** Different lowercase letters indicate significant differences between different incubation times in the same treatment (*P* < 0.05). CK, control (circles); FO1, combination of 60% chemical fertilizer with organic manure of 150 kg/ha (triangles); FO2, combination of 60% chemical fertilizer with organic manure of 300 kg/ha; FO3, combination of 60% chemical fertilizer with organic manure of 450 kg/ha (squares). **(B)** CK, control (filled circles); FO1, FOL combination of 60% chemical fertilizer with organic manure of 150 kg/ha (filled squares); FO2, combination of 60% chemical fertilizer with organic manure of 300 kg/ha (filled triangles); FO3, combination of 60% chemical fertilizer with organic manure of 450 kg/ha (filled diamonds); PC, principal component.

No significant difference was found in *AWCD* values at 24 h of incubation in different soil treatments, indicating that the use of carbon sources by soil microorganisms was weak. However, the *AWCD* values increased sharply from 24 to 144 h, reaching their highest value at 144 h (*P* < 0.05). The *AWCD* values continued to increase slowly from 144 to 240 h but without significant differences (Figure 1A). This indicated that the soil microbial activity and carbon source utilization ability were strongest at the “inflection point” of 144 h. Of all treatments, CK showed the lowest *AWCD* values, and the highest values were observed in FO3 (*P* < 0.05).

The PCA was used to analyze the utilization of 31 carbon sources as substrates at 144 h by soil microbes (Figure 1B). The two principal component factors (PC1 and PC2), acquired from 31 carbon sources, could explain 39.6 and 16.8% of the variables, respectively. The scores of CK and FO treatments were well separated in the PC1 axis. FO3 was clearly separated from other treatments in the PC2 axis. As shown in Table 4, based on the eigenvector coefficients (loadings) of greater than 0.60 for PC1 and PC2, there were 18 types of carbon sources significantly in PC1, including four carbohydrates, four amino acids, three carboxylic acids, three polymers, two amines, and two phenolic acids, among which carbohydrates and amino acids accounted for 44.4% and contributed the most to PC1. There were five types of carbon sources significantly present in PC2, including carbohydrates (3), carboxylic acids (1), and phenolic acids (1), among which carbohydrates contributed the most, accounting for 60%. In short, carbohydrates and amino acids were the most sensitive carbon sources for soil microorganisms. Additionally, the dominant carbon sources with PC1 and PC2 were D-Mannitol (carbohydrates) and β-Methyl D-Glucoside (carbohydrates), respectively (Table 4). The above results show that organic fertilizer substitution treatments improved the carbohydrate and amino acid utilization abilities of soil microorganisms in rain-fed wheat fields.

**Table 4:** Main carbon sources and corresponding PC loadings in different treatments.

**Main carbon sources types**	**PC1**	**Main carbon sources types**	**PC2**
**Carbohydrates**		**Carbohydrates**	
D-Mannitol	0.892	β-Methyl-D-glucoside	0.924
Glucose-1-phosphate	0.880	I-erythritol	−0.795
D, L-a-glycerol	−0.855	D-Galactonic acid y-lactone	0.647
a-D-Lactose	0.720	**Carboxylic acids**	
**Amino acids**		y-Hydroxybutyric acid	−0.670
L-Threonine	−0.832	**Phenolic acids**	
L-Phenylalanine	−0.800	2-Hydroxybenzoic acid	0.602
L-Asparagine	0.763		
L-Serine	0.712		
**Carboxylic acids**			
a-Ketobutyric acid	−0.827		
Pyruvic acid methyl ester	0.784		
Itaconic acid	0.755		
**Polymers**			
Glycogen	−0.856		
80Tween 80	0.769		
a-Cyclodextrin	0.663		
**Amines**			
Phenylethyl-amine	−0.795		
Putrescine	0.696		
**Phenolic acids**			
2-Hydroxybenzoic acid	−0.695		
4-Hydroxybenzoic acid	0.672		

**Figure 2:**
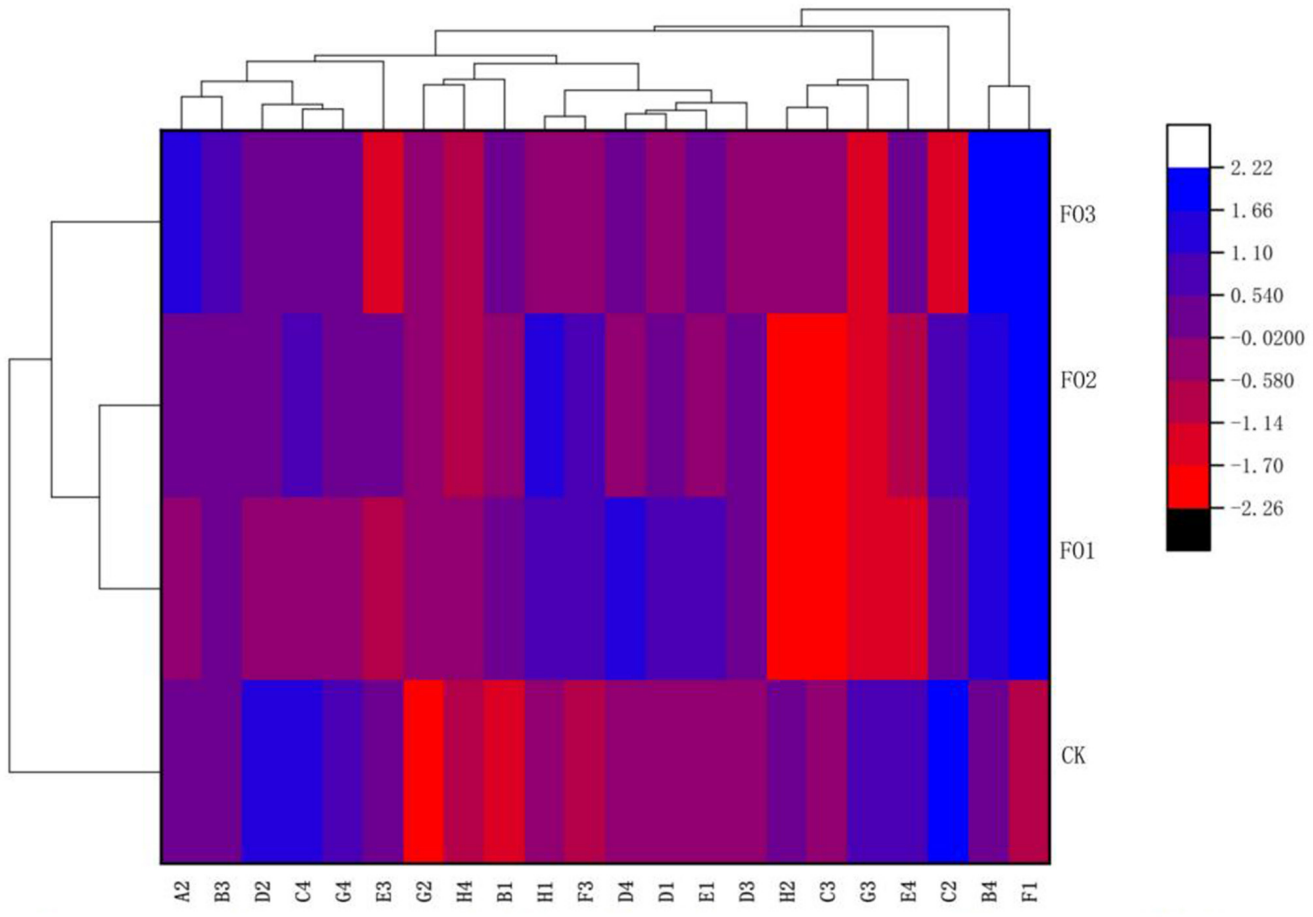
Cluster diagram of soil microbial carbon sources utilization under different treatments. The carbon sources are listed on the *X*-axis and treatments on the *Y*-axis. For the heat map, the left side of the cluster tree is a treatment cluster tree and the above cluster tree is the carbon sources duster tree. A2, represents β-methyl-D-glucoside; B3, D-galacturonic acid; D2, D-mannitol; C4, L-phenylalanine; G4, phenylethyl-amine; E3, y-hydroxybutyric acid; G2, Glucose-1-Phosphate; H4, Putrescine, B1, pyruvic acid methyl ester; H1, a-D-Lactose; F3, itaconic acid; D4, L-Serine; D1, Tween 80; E1, a-Cyclodextrin; D3, 4-hydroxybenzoic acid; H2, D,L-a-glycerol; C3, 2-hydroxybenzoic acid; G3, a-ketobutyric acid; E4, L-threonine; C2, I-Erythritol; B4, L-asparagine; Fl, represents Glycogen. While CK, shows control; FO1, combination of 60% chemical fertilizer with organic manure of 150 kg/ha; FO2, combination of 60% chemical fertilizer with organic manure of 300 kg/ha; and corresponding FO3, combination of 60% chemical fertilizer with organic manure of 450 kg/ha. The numbers for different colors of the columns represent the abundance of the genera or genes in different samples.

Furthermore, through the cluster analysis of the utilization ability of the 22 types of carbon sources mentioned above on the basis of the *AWCD* values (Figure 2), three clusters were obtained: one cluster was observed in FO1 and FO2, and two clusters were found in CK and FO3, respectively. The results showed that soil microorganisms' carbon source utilization types for organic fertilizer and chemical fertilizer treatments differed significantly. For example, soil microbes in the organic fertilizer substitution treatments could utilize more F1 (glycogen) than those in CK, but in CK, a larger amount of C2 (I-Erythritol), D2 (D-Mannitol), and C4 (L-Phenylalanine) was used by soil microorganisms. In addition, a larger amount of A2 (β-Methyl D-Glucoside) and B4 (L-Asparagine) was utilized by soil microorganisms in FO3, but more D4 (L-Serine) and H1 (α-D-Lactose) were used by soil microorganisms in FO1 and FO2, respectively (Figure 2).

### 3.3. Soil bacterial community composition and PICRUSt functional predictive analysis

**Figure 3:**
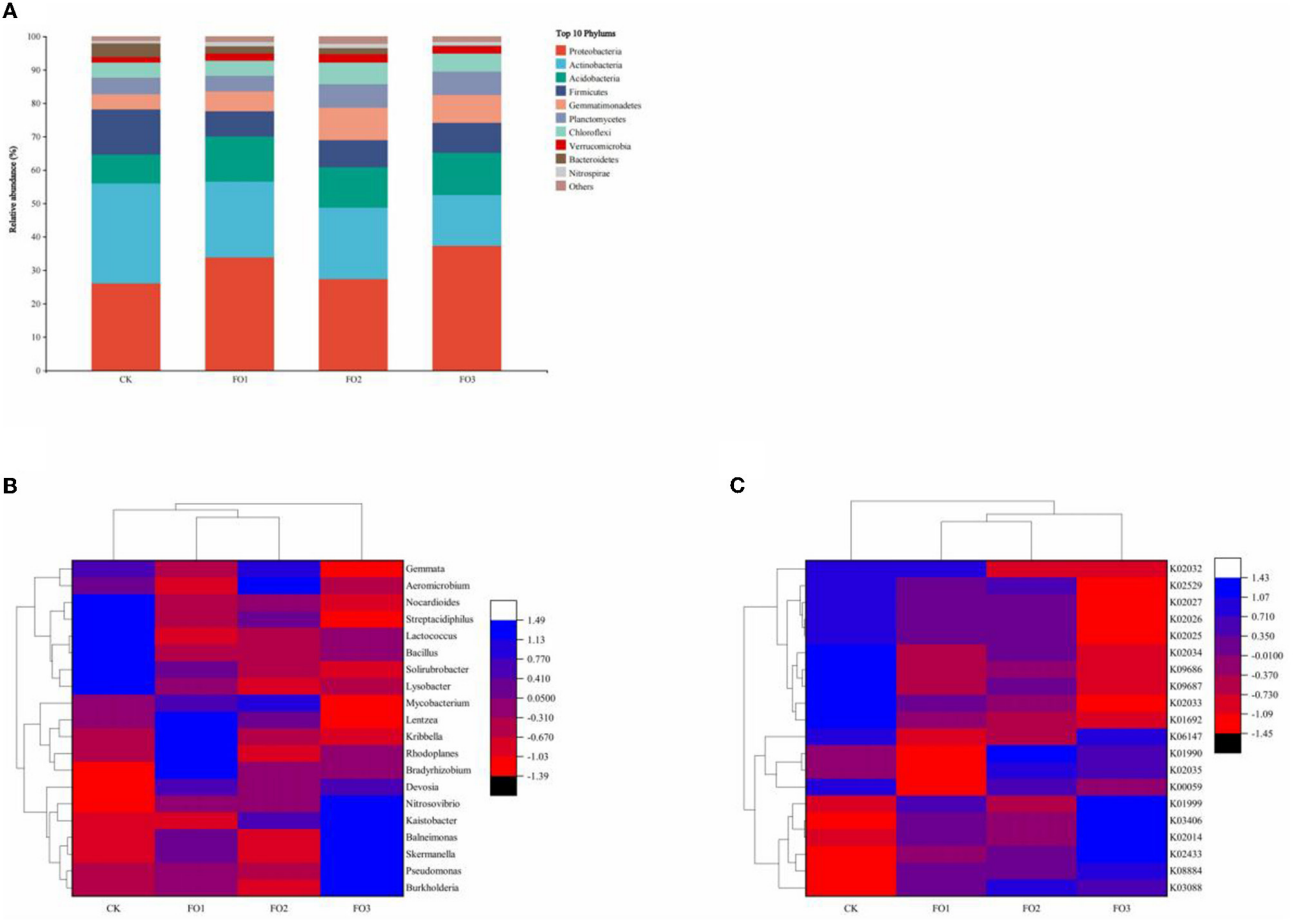
Changes in the relative abundance of soil bacteria at the phylum level **(A)** among different treatments. The heatmap of relative abundances of the top 20 identified bacteria at the genus level **(B)** and bacterial community KEGG Orthologs (KOs) for different soil samples **(C)**. CK, shows control; FO1, combination of 60% chemical fertilizer with organic manure of 150 kg/ha; FO2, combination of 60% chemical fertilizer with organic manure of 300 kg/ha; FO3, combination of 60% chemical fertilizer with organic manure of 450 kg/ha. Numbers of different colors of the columns represent the abundance of the genera or genes in different samples.

Figure 3A depicts the relative abundance of the top 10 bacteria at the phylum level in all treatments. The most dominant bacterial phyla were Proteobacteria, Actinobacteria, Acidobacteria, Firmicutes, and Gemmatimonadetes. Proteobacteria were abundant in all treatments (>25%). Additionally, its relative abundance was higher in organic fertilizer treatments than in CK. The relative abundance of Actinobacteria was the highest (30%) in the CK. The cluster analysis of relative abundance at the genus level of bacteria (Figure 3B) was divided into two main categories, namely FO3 and other treatments. FO1 and FO2 were further clustered into one category. Among the top 20 abundantly identified soil bacterial genera, 10 genera (*Burkholderia, Skermanella, Lysobacter, Kaistobacter, Balneimonas, Bradyrhizobium, Rhodoplanes, Pseudomonas, Nitrosovibrio, and Devosia*) belonged to Proteobacteria, seven genera (*Kribbella, Solirubrobacter, Aeromicrobium, Mycobacterium, Streptacidiphilus, Lentzea, and Nocardioides*) belonged to Actinobacteria, two genera (*Bacillus* and *Lactococcus*) belonged to Firmicutes, and one genus (*Gemmata*) belonged to Planctomycetes. In CK, *Nocardioides, Streptacidiphilus, Lactococcus, Bacillus, Solirubrobacter*, and *Lysobacter* were dominant and significantly higher than in other treatments. In FO3, the relative abundance of *Nitrosovibrio, Kaistobacter, Balneimonas, Skermanella, Pseudomonas*, and *Burkholderia was* significantly increased. For example, the relative abundance of *Nitrosovibrio* followed the following order: FO3 > FO2 > FO1 > CK. These results indicated that the organic fertilizer substitution for a partial chemical fertilizer regime significantly affected soil bacterial community composition in rain-fed wheat fields.

The relative abundance of KEGG Orthologs (KOs) of the bacterial community exhibited significant variations among treatments, and different soil samples were grouped into three clusters (Figure 3C); CK and FO3 treatments were two separate clusters, and FO1 was clustered with FO2. In CK, K02034 (the peptide/nickel transport system permease protein), K09686 (the antibiotic transport system permease protein), K09687 (the antibiotic transport system ATP-binding protein), and K01692 (the enoyl-CoA hydratase) were dominant compared to other treatments. FO3 significantly increased the relative abundance of K01999 (the branched-chain amino acid transport system substrate-binding protein), K03406 (the methyl-accepting chemotaxis protein), K02014 (the iron complex outer membrane receptor protein), and K02433 [the aspartyl-tRNA (Asn)/glutamyl-tRNA (Gln)]. We found that the relative abundance of K02433 also followed the order FO3 > FO2 > FO1 > CK, which is consistent with the change trends of the relative abundance of *Nitrosovibrio*. Similarly, the relative abundance changes of K03406 and K02014 were consistent with those of *Skermanella* and *Burkholderia*.

### 3.4. Correlation analysis between soil carbon source utilization, KEGG Orthologs, soil nutrients, and wheat yield under CK and FO3 treatments

**Figure 4:**
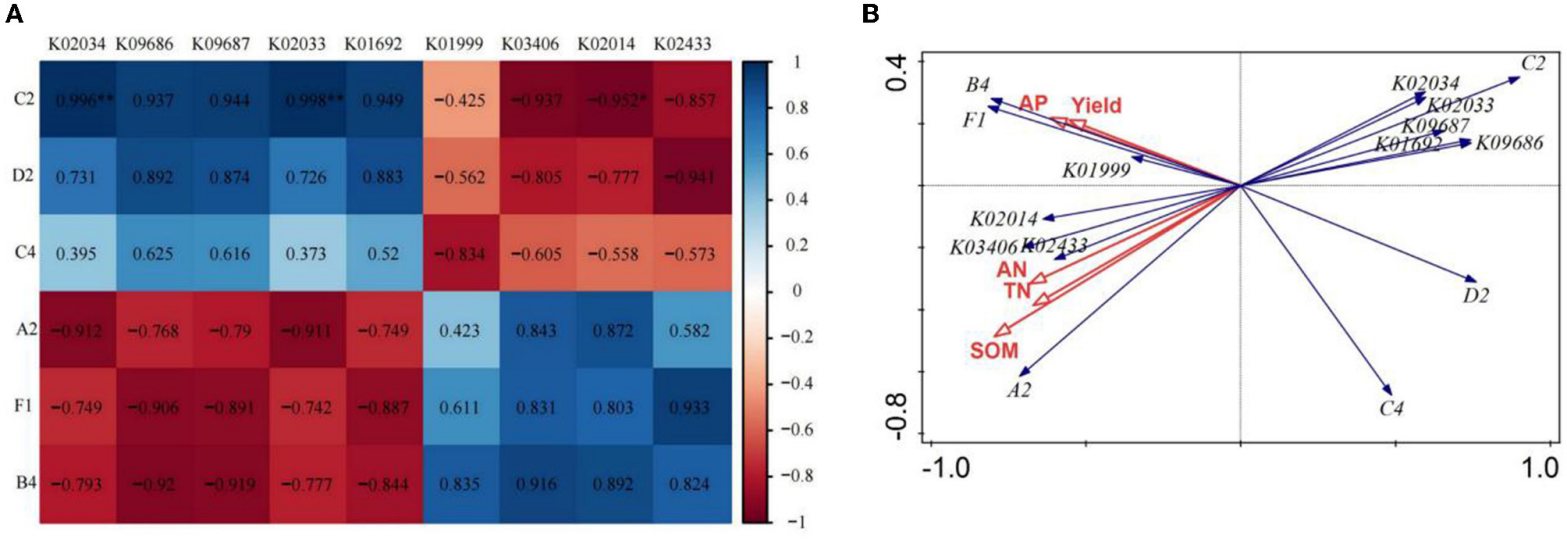
Correlation analysis of dominant soil carbon metabolism and dominant KEGG Orthologs (KOs) in rain-fed wheat field **(A)**; redundancy analysis of soil nutrient and yield with dominant carbon source utilization and dominant bacteria KEGG Orthologs **(B)**. A2, represents β-Methyl-D-Glucoside; D2, D-Mannitol; C4, L-phenylalanine; C2, I-Erythritol; B4, L-asparagine; Fl, represents glycogen. TN, total nitrogen; AN, available nitrogen; AP, available phosphorus; SOM, soil organic matter. While CK, shows control; FO1, combination of 60% chemical fertilizer with organic manure of 150 kg/ha; FO2, combination of 60% chemical fertilizer with organic manure of 300 kg/ha; and corresponding FO3, combination of 60% chemical fertilizer with organic manure of 450 kg/ha. The numbers for different colors of the columns represent the abundance of the genera or genes in different samples.

Based on the above analysis, we chose the CK and FO3 treatments to conduct the correlation analysis between soil carbon source utilization, KEGG Orthologs (KOs), soil nutrients, and wheat yield. As shown in Figure 4A, the utilization of C2 (I-Erythritol) by soil microorganisms was significantly negatively correlated with K02014 (iron complex *in vitro* membrane receptor protein). However, it showed a very significant positive correlation with K02034 (permease protein of the peptide/nickel transport system) and K02033 (peptide/nickel transport system permease protein). The utilization of D2 (D-Mannitol) by soil microorganisms was positively correlated with K09686 (the antibiotic transport system permease protein) and K09687 (the antibiotic transport system ATP-binding protein) and was negatively correlated with K02433 [the aspartyl-tRNA (Asn)/glutamyl-tRNA (Gln)]. The utilization of A2 (β-Methyl-D-Glucoside) by soil microorganisms was negatively correlated with K02034 and K02033 and was positively correlated with K02014. The utilization of F1 (glycogen) and B4 (L-Asparagine) by soil microorganisms was negatively correlated with K09686 and K09687 and positively correlated with K02433.

The redundancy analysis of wheat yield, soil nutrients, soil microbial carbon source utilization, and KEGG Orthologs (KOs; Figure 4B) showed that the utilization of C2 (I-Erythritol), D2 (D-Mannitol) and C4 (l-Phenylalanine) by soil microorganisms was negatively correlated with wheat yield, SOM, TN, AN, AP, and the utilization of A2 (β -Methyl-D-Glucoside), B4 (L-Asparagine) and F1 (glycogen) by soil microorganisms. It was also found that wheat yield, SOM, TN, AN, and AP were negatively correlated with K02033, K02034, K09687, and K09686 but were positively correlated with K01999, K02014, K03406, and K02433.

## 4. Discussion

### 4.1. Changes of soil nutrients, wheat yield, partial productivity of fertilizer, soil microbial carbon source utilization, and KEGG Orthologs in a rain-fed wheat field

Soil microorganisms had corresponding nutrient preferences, which may alleviate nutrient limitations (Cui et al., 2018). The organic fertilizer application could increase the contents of carbohydrates and amino acids in soil (Xu et al., 2003) to maintain the soil microbial groups metabolizing these two types of carbon sources (Mao et al., 2020; Yang et al., 2020). The organic fertilizer application promoted the metabolism of amino acids (L-Asparagine) by soil microorganisms (Kumar et al., 2017). In this study, we found that the sensitive carbon sources for soil microorganisms that were greatly affected were mainly carbohydrates and amino acids. The carbon sources utilized by soil microorganisms were mainly I-Erythritol (C2), D-mannitol (D2), and L-Phenylalanine (C4) in CK, while they were mainly β-methyl D-glucoside (A2), L-Asparagine (B4), and glycogen (F1) in FO3. This indicated that applying organic fertilizer changed soil microorganisms' ability to utilize different carbon sources (Tang et al., 2019).

Previous studies have discovered that after the addition of exogenous p-hydroxybenzoic acid (pHA) to the soil, the contents of AN, AP, and SOM in the soil reduced (Harper, 1977; Iii and Stuart, 1995; Zavarzina et al., 2019), which promoted the growth of nitrogen-fixing bacteria, ammonifying bacteria, denitrifying bacteria, and nitroso bacteria in the soil and inhibited the growth of nitrifying bacteria (Gu et al., 2020). Another study showed that the *Nitrovibrio* genus was enriched in organic fertilizer treatments during the wheat season (Shi et al., 2020). This study showed that organic fertilizer substitution treatments could promote the use of phenols (4-hydroxybenzoic acid, the main chemical component of root exudates of wheat and other crops, has strong allelopathy and may lead to lower crop yield) by soil microorganisms and increase the contents of TN, SOM, AN, and AP in soil and the relative abundance of *Nitrovibrio*. Therefore, organic fertilizer substitution for chemical fertilizer could promote the metabolism of these carbon sources, thereby improving soil nutrients and wheat yield (Han et al., 2021a; Liu et al., 2021). The soil microorganisms' utilization degree of β-Methyl-D-Glucoside, L-asparagine, and glycogen was higher in the FO3 treatment. This might be related to the highest relative abundance of *Skermanella*, which can utilize D-Glucose, D-Mannitol, lactose, sucrose, aspartate, and pyruvate (Subhash et al., 2017).

This study shows that the relative abundance of K02014 (iron complex outer membrane receptor protein) is consistent with *Burkholderia* (Lin et al., 2019), which is known for being involved in several iron uptake pathways, including siderophore production and the ability to absorb heme in infected hosts (Pongmala et al., 2022). K02433 represents an aminoacyl tRNA synthetase that can specifically recognize aspartic acid and glutamyl amine and form aminoacyl tRNA with the corresponding tRNA. Ammonium is the main nitrogen source of plants, and it is assimilated into asparagine through the catalytic reaction of asparagine synthetase (GLN), which then forms aspartic amino acid through different amino acid transferases to complete the recycling in ammonia sources of different forms (Cid et al., 2022). In this study, the abundance of related enzyme genes in the above amino acid metabolism process showed clear enrichment in FO3. Therefore, it was speculated that organic fertilizer could accelerate the conversion of ammonium nitrogen into glutamine and further into various amino acids after entering the soil environment, effectively improving the utilization rate of ammonium nitrogen uptake by crops. Indeed, the degree of utilization of B4 (L-Asparagine) by soil microorganisms in FO3 was significantly higher than that of other carbon sources. Organic fertilizer can accelerate the abundance of *Pseudomonas* and promote the formation of biofilms between specific bacterial groups, which is conducive to plant disease resistance (Chen et al., 2023).

Moreover, the increased abundance of *Pseudomonas also* promoted soil nitrogen metabolism (Fu et al., 2023). The enrichment of K03406 (the methyl-accepting chemotaxis protein) in FO3 is also important evidence. The results showed that the interaction between microorganisms and soil was greatly affected by organic fertilizer.

The results also showed that the partial productivity of fertilizer was significantly increased under the organic fertilizer substitution treatments, indicating that the utilization efficiency of fertilizer can be increased by optimizing the application of fertilizer (Liu et al., 2018).

### 4.2. Relationship between soil carbon source utilization, KEGG Orthologs, soil nutrients, and wheat yield under CK and FO3 treatment

Redundancy analysis of soil nutrients, wheat yield, soil microbial carbon sources, and KEGG Orthologs (KOs) showed that the microbial utilization of A2 (β-Methyl-D-Glucoside), B4 (L-Asparagine), and F1 (glycogen) carbon sources was positively correlated with KOs (K01999, K02014, K03406, K02433), soil nutrients (SOM, TN, AN, and AP), and wheat yield.

In particular, soil microbial utilization of B4 and F1 showed a significantly positive correlation with K01999, wheat yield, and AP. K01999 (the branched-chain amino acid transport system is a substrate-binding protein). Branched-chain amino acids are a class of essential amino acids with various physiological and metabolic roles (Virlouvet et al., 2011). The carbon skeleton required for amino acid synthesis comes from glycolysis, photosynthesis, the oxidative pentose phosphate pathway, and the tricarboxylic acid cycle (TCA). Therefore, the synthesis of amino acids is the hub of carbon and nitrogen metabolism (Xin et al., 2019). B4 (L-Asparagine) is one of the metabolites of the TCA cycle and the related amino acid metabolism pathway (Baslam et al., 2020; Han et al., 2021b). Phosphorus-solubilizing bacteria can transform insoluble phosphorus, which is difficult for plants to absorb and utilize, into effective forms and improve the utilization efficiency of soil phosphorus (Kalayu, 2019). *Pseudomonas* is agricultural soil's main phosphorus-solubilizing bacteria (PSB) (Wang et al., 2022). Soil AP content was closely related to soil bacterial community and wheat yield, indicating that soil phosphorus was an important factor driving soil microbial community and wheat yield changes (Chen et al., 2021).

The utilization of an A2 carbon source by soil microorganisms was significantly positively correlated with K02433, K02014, and SOM. This showed that the application of organic fertilizer could increase SOM content, improve soil microbial diversity, stimulate soil microbial activity (Li et al., 2020), cause organic carbon decomposition, and then cause dramatic changes in soil carbon sources (Haiming et al., 2020). In this way, plants could uptake and utilize a large number of available nutrients (Zhao et al., 2021), thus increasing the plant biomass, and the high plant biomass will secrete more simple organic matter to further improve microbial activity.

## 5. Conclusion

In the present study, organic fertilizer substitution treatments affected carbon source utilization by soil microorganisms and microbial community composition. Particularly, FO3 treatment improved the utilization of carbohydrates and amino acids by soil microorganisms and enriched bacterial genera (*Skermanella, Pseudomonas*, and *Burkholderia*). Furthermore, when subjected to FO3 treatment, the dominant KOs (K01999, K03406, K02014, and K02433) were strongly correlated with soil nutrients, contributing to soil nutrient cycling and crop productivity. In conclusion, adding organic fertilizer was crucial for improving soil quality, promoting agricultural production, regulating soil microbial metabolic capacity, and changing bacterial community composition. In addition, FO3 treatment is recommended as an appropriate fertilization method in this study.

## Data availability statement

The datasets presented in this study can be found in online repositories. The names of the repository/repositories and accession number(s) can be found below: NCBI - PRJNA948916.

## Author contributions

ZY and XL conceived and designed the experiments. XL and WL conducted the experiments. WY, JC, and AA analyzed the data. MS and ZG contributed analysis tools. XL, AA, and ZY wrote the article. All authors contributed to the article and approved the submitted version.
